# Clinicopathological and prognostic significance of high circulating lymphocyte ratio in patients receiving neoadjuvant chemotherapy for advanced gastric cancer

**DOI:** 10.1038/s41598-018-24259-5

**Published:** 2018-04-18

**Authors:** Yang Li, Yao Wei, Qi He, Xulin Wang, Chaogang Fan, Guoli Li

**Affiliations:** 1 Division of Digestive Surgery, Xijing Hospital, Fourth Military Medical University, 127 West Changle Road, 710032 Xi’an, Shaanxi China; 2 Research Institute of General Surgery, Jinling Hospital, School of Medicine, Nanjing University, 305 Zhongshan Eastern Road, Nanjing, 210002 China; 3 The First Affiliated Hospital of Soochow University, Department of Medicine, Emergency and Critical Care Medicine, Suzhou, 215003 China

## Abstract

This study was designed to investigate the prognostic value of circulating blood cell counts and subsets for patients with advanced gastric cancer (AGC) treated with neoadjuvant chemotherapy (NAC) and the factors determining pathological complete response (pCR). In 112 patients with AGC, we retrospectively examined the ratios of lymphocyte, monocyte, and neutrophil during and after NAC before surgery, and the percentages of CD3+, CD3+ CD4+, CD3+ CD8+ and CD4+/CD8+ lymphocytes as well. We also investigated their associations with the pCR rate and overall survival (OS). The ratios of lymphocyte taken before and after NAC were significantly greater in forty-four pCR cases than that in sixty-eight non-pCR cases. During NAC, the proportion of lymphocyte and the percentages of CD3+, CD3+ CD4+, and CD3+ CD8+ lymphocytes were dramatically increased in pCR group. The lymphocyte ratio showed an independent association with pCR by multivariate analysis and maintained at a relatively high level in pCR cases. By mean of 31.53% lymphocyte ratio before-NAC and 41.68% after-NAC, cases with high lymphocyte ratio showed significantly better outcome in OS. High circulating lymphocyte ratios, both before and after NAC, are positively associated with pCR and improved OS in advanced gastric cancer, which may be considered as a new prognostic biomarker.

## Introduction

Gastric cancer remains one of the most common malignancies around the world and causes 499,000 cancer-related deaths each year in China^1–4^. Moreover, a majority of gastric cancer patients (80–90%) in China are diagnosed at advanced stages with extensive regional lymph node involvement and/or invasion of adjacent structures in first medical consultation^5,6^. Neoadjuvant chemotherapy (NAC) with many clinical advantages is a promising strategy and currently accepted as an effective treatment for various malignant diseases, including ovarian, head and neck cancer and extremity tumors^7^. However, not all AGC patients benefit from NAC: studies not only suggest that the overall response rate to NAC is less than 50% but also highlight that nearly 15% of patients undergoing NAC show risks of tumor progression^8,9^. Additionally, the 5-year survival rate of AGC patients remains at 45–50% even after comprehensive strategies including chemotherapy and surgery^10–12^. The pathological complete response (pCR) to NAC has been reported to correlate with a favorable long-term outcome^13–16^. However, it turns out rarely in patients with AGC, despite the application of various combined chemotherapy regimens. Therefore, it would be advantageous to determine which factor could predict the efficacy of NAC and to identify what kind of patients might gain pCR and a better long-term outcome.

The immune response to gastric cancer is complex, involving the interaction of several cell types of the immune system, which plays a significant role in the progression of gastric cancer. Several studies have indicated that many patients with gastric cancer have various scales of immunological impairment, including decreased cellular immunity^17,18^. Besides, the prevalence of suppressor cells may prove to be a decisive factor for poor outcomes because regulatory T cells restrain the antitumor activity of cytotoxic T cells^19,20^. A recent meta-analysis study^21^ has shown that the elevated platelet to lymphocyte ratio could be a significant prognostic biomarker for poor overall survival (OS) in patients with gastric cancer. Since blood cell counts in peripheral blood were considered to reflect the immunological function in AGC patients, we have endeavored to determine whether the values of lymphocytes before or after NAC may serve as new parameters predicting pCR to NAC. We also examined the laboratory data of white blood cell and lymphocyte subpopulation during and after NAC period before surgery, which may reflect systemic responses against tumor cells damaged by NAC. Besides, the correlation between clinical parameters and outcomes was examined to determine the potential prognostic impact of lymphocyte on OS.

## Results

### Patients characteristics

**Figure 1:**
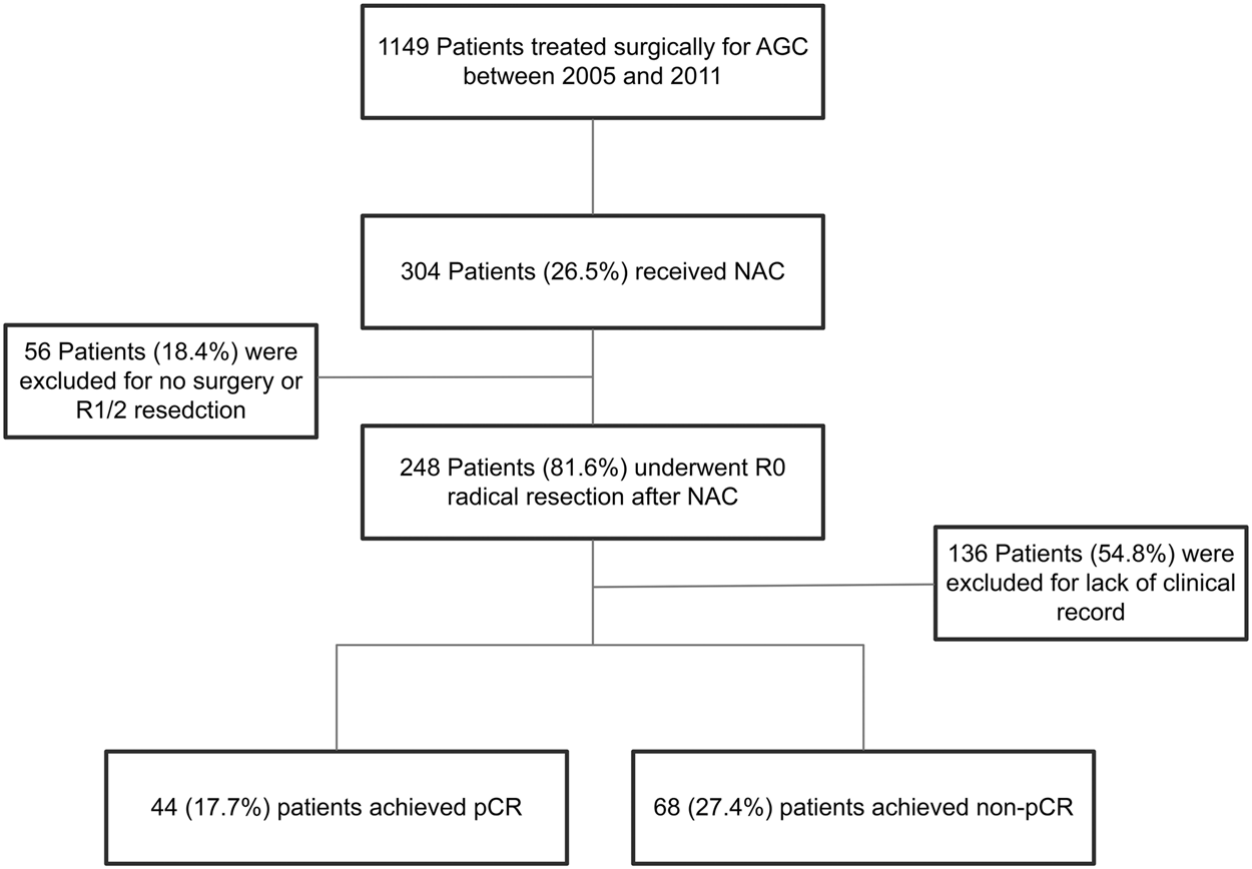
Patient study group CONSORT diagram.

From January 2005 to December 2011, 1149 patients who underwent surgical treatment for AGC were reviewed in our institution. Among them, 304 cases (26.5%) received NAC for AGC. R0 radical resection was performed in 248 (81.6%) patients, with 44 cases of them (17.7% of NAC treatment cases who underwent R0 resection) being demonstrated no residual tumor on final pathology and defined as pCR. The remaining 204 patients (82.2% of NAC treatment cases who underwent R0 resection) were identified as non-pCR after surgery, from which 68 cases (27.4%) were included, and 136 cases (54.8%) were excluded for lack of clinical records (Fig. 1).

### Clinical and pathological factors

The baseline characteristics of all patients in pCR group and non-pCR group are given in Table 1. There were no differences in all clinical factors, including age, gender, Borrmann type, differentiation, histological type, clinical stage (TNM), carcinoembryonic antigen (CEA), and albumin (ALB) between pCR group and non-pCR group.

**Table 1:** Correlation between clinical and pathological factors before NAC and pathological response in patients.

Variable	Non-pCR (n = 68)	pCR (n = 44)	*P* value
Age (years)	58.57 ± 9.59	60.89 ± 7.89	0.185
Gender			
Male	46	34	0.293
Female	22	10	
Primary tumor			
Gross tumor type (Borrmann)			0.733
I	3	2	
II	33	17	
III	21	15	
IV	11	10	
Differentiation			0.340
High-medium	24	15	
Low	40	29	
Undifferentiated	3	0	
Histological type			0.982
Adenocarcinoma	54	36	
Mucinous cancer	10	6	
Signet ring cancer	3	2	
Clinical stage (TNM)			
T Classification			0.611
T3	12	6	
T4	56	38	
N Classification			0.317
N1	15	12	
N2	11	11	
N3	42	21	
Stage			0.671
IIIA	18	15	
IIIB	15	8	
IIIC	35	21	
CEA			0.321
≤5 ng/ml	51	11	
>5 ng/ml	17	33	
ALB (g/L)	39.34 ± 5.39	39.25 ± 4.79	0.927

CEA carcinoembryonic antigen; ALB albumin.

### Lymphocyte ratio was associated with the pCR rate and OS

Blood cell counts recorded before and after NAC were compared between pCR group and non-pCR group, respectively (Table 2). There were no statistical differences between the two groups in some blood cell data before NAC, including the ratios of monocyte and neutrophil and the percentages of CD4+/CD8+, CD8+ CD3+, CD3+ and CD4+ CD3+ lymphocytes. And after NAC, there were no differences between the two groups in the ratios of monocyte and neutrophil and the percentages of CD4+/CD8+ and CD4+ CD3+ lymphocytes. However, both before and after NAC, the ratios of lymphocyte were significantly higher in pCR group than that in non-pCR group (31.53 ± 5.01 vs. 25.68 ± 8.93 *p* < 0.01, 41.68 ± 8.40 vs. 32.54 ± 11.01 *p* < 0.01, respectively). Also, the percentages of CD3+ and CD8+ CD3+ lymphocytes were statistically increased in pCR group after NAC (69.36 ± 13.41 vs. 61.72 ± 13.37 *p* < 0.05, 32.56 ± 18.85 vs. 24.38 ± 9.52 *p* < 0.01, respectively). Additionally, the level of platelet before NAC tended to be lower in pCR group, but marked differences weren’t noted.

**Table 2:** Blood cell data and pathological response in AGC patients

Blood Cell data	Non-pCR (n = 68)	pCR (n = 44)	*P* value
PLT (×10^3^ mm3)	241.91 ± 109.07	222.44 ± 64.49	0.127
Pre-NAC (%)			
CD4+/CD8+	1.78 ± 1.08	1.74 ± 0.84	0.843
CD8+CD3+	21.20 ± 7.86	18.93 ± 6.49	0.063
CD3+	53.29 ± 12.39	49.54 ± 14.18	0.079
CD4+CD3+	32.12 ± 9.42	30.64 ± 11.55	0.141
lymphocyte	25.68 ± 8.93	31.53 ± 5.01	**0.001**
monocyte	6.61 ± 1.77	7.19 ± 1.92	0.120
neutrophil	64.47 ± 9.86	63.56 ± 10.07	0.755
After-NAC (%)			
CD4+/CD8+	1.88 ± 1.18	1.63 ± 1.11	0.394
CD8+CD3+	24.38 ± 9.52	32.56 ± 18.85	**0.001**
CD3+	61.72 ± 13.37	69.36 ± 13.41	**0.031**
CD4+CD3+	37.17 ± 9.77	36.79 ± 12.94	0.631
lymphocyte	32.54 ± 11.01	41.68 ± 8.40	**0.001**
monocyte	51.67 ± 15.13	51.15 ± 11.69	0.455
neutrophil	11.73 ± 5.26	11.66 ± 3.64	0.074

AGC advanced gastric cancer; PLT platelet; NAC neoadjuvant chemotherapy.

**Figure 2:**
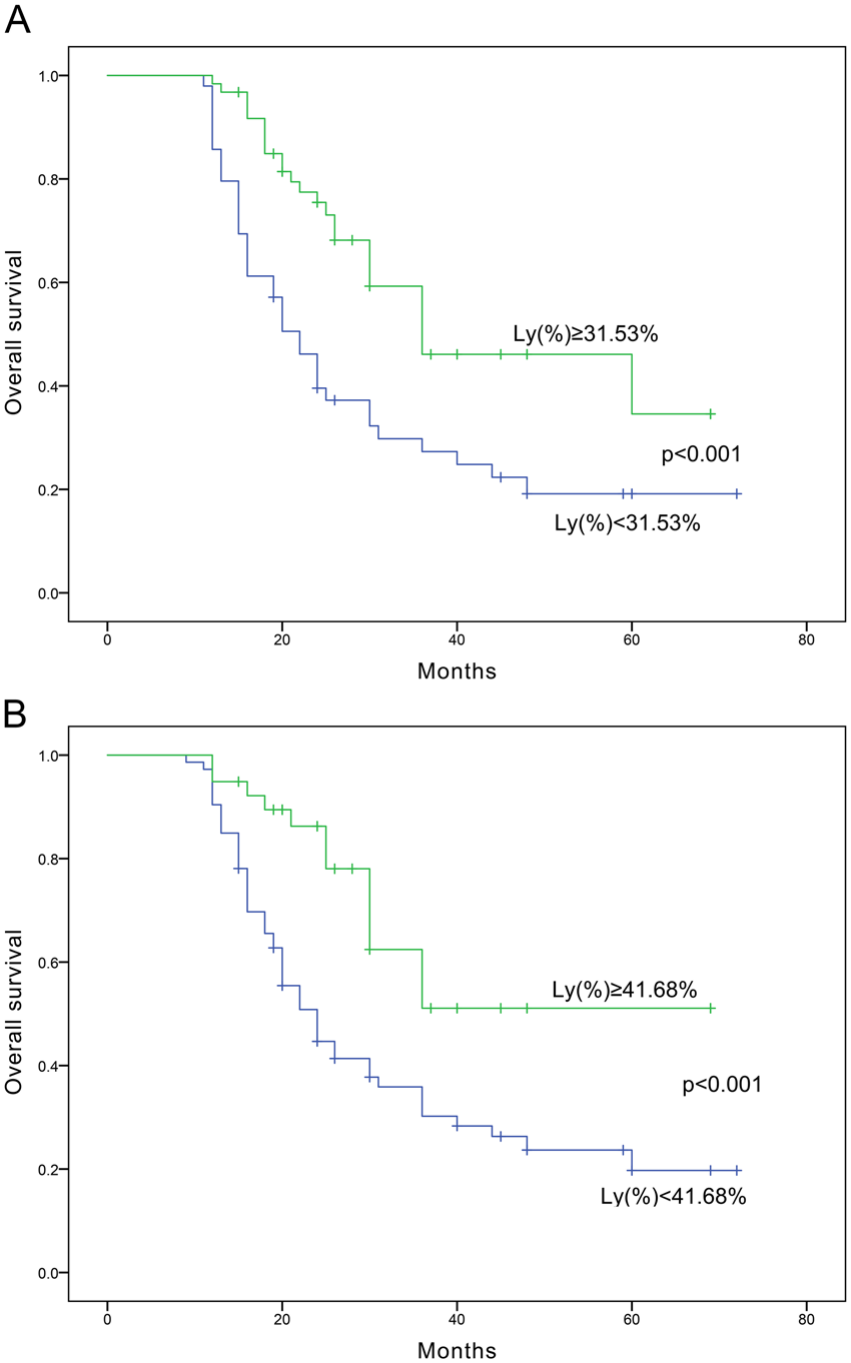
Overall survival of the patients with high and low lymphocyte ratios taken before and after NAC.

As shown in Table 3, multivariate analysis revealed that lymphocyte ratios, both before and after NAC, showed independent correlation with the pCR rate (*p* = 0.015 and *p* = 0.006, respectively). In addition, after divided into high or low lymphocyte ratio groups by the mean of 31.53% before-NAC or by the mean of 41.68% after-NAC, patients in high lymphocyte ratio group showed significantly better outcomes in median OS (high group vs. low group before NAC: 36 vs. 22 months, *p* < 0.001; high group vs. low group after NAC: 44 vs. 24 months, *p* < 0.001, respectively) (Fig. 2A,B).

**Table 3:** Multivariate analysis of pCR rate.

Variable	Odds (95% CI)	*P* value
Tumor size	1.019 (0.834–1.244)	0.854
Clinical stage	1.150 (0.684–2.041)	0.634
% lymphocyte before NAC	0.002 (0.001–0.147)	**0.015**
% CD8+CD3+ before NAC	1.215 (0.840–1.757)	0.302
% CD3+ before NAC	0.939 (0.659–1.336)	0.725
% CD4+CD3+ before NAC	1.075 (0.759–1.525)	0.683
% lymphocyte after NAC	0.001 (0.000–0.116)	**0.006**
% CD8+CD3+ after NAC	0.943 (0.715–1.244)	0.676
% CD3+ after NAC	0.988 (0.754–1.296)	0.932
% CD4+CD3+ after NAC	0.967 (0.735–1.273)	0.812

NAC neoadjuvant chemotherapy; pCR pathological complete response.

### Leukocyte subpopulation and lymphocyte subsets during NAC

**Figure 3:**
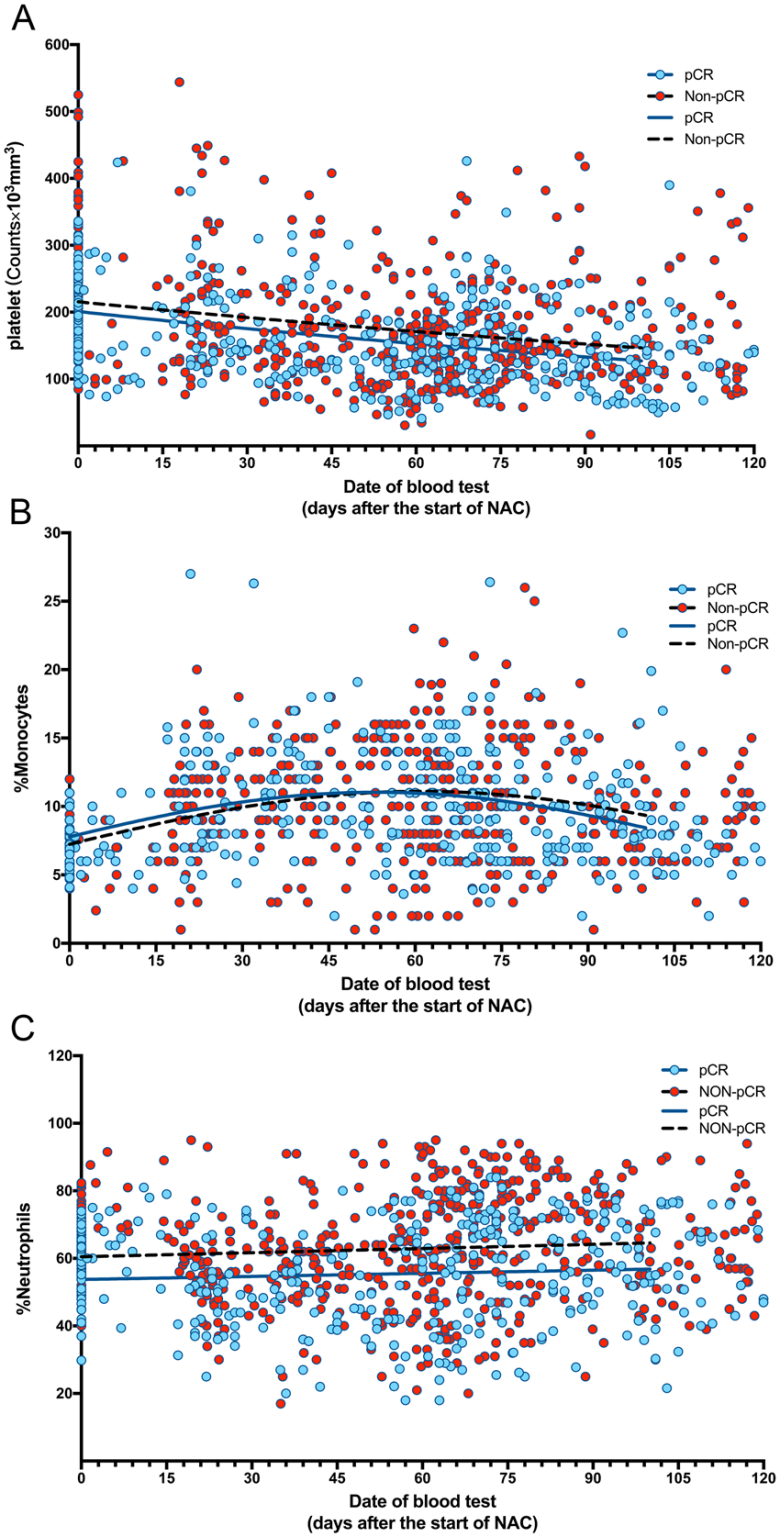
Changes in the count of platelet (**A**), the ratio of monocytes (**B**) and neutrophils (**C**) in peripheral blood samples during entire NAC treatment.

**Figure 4:**
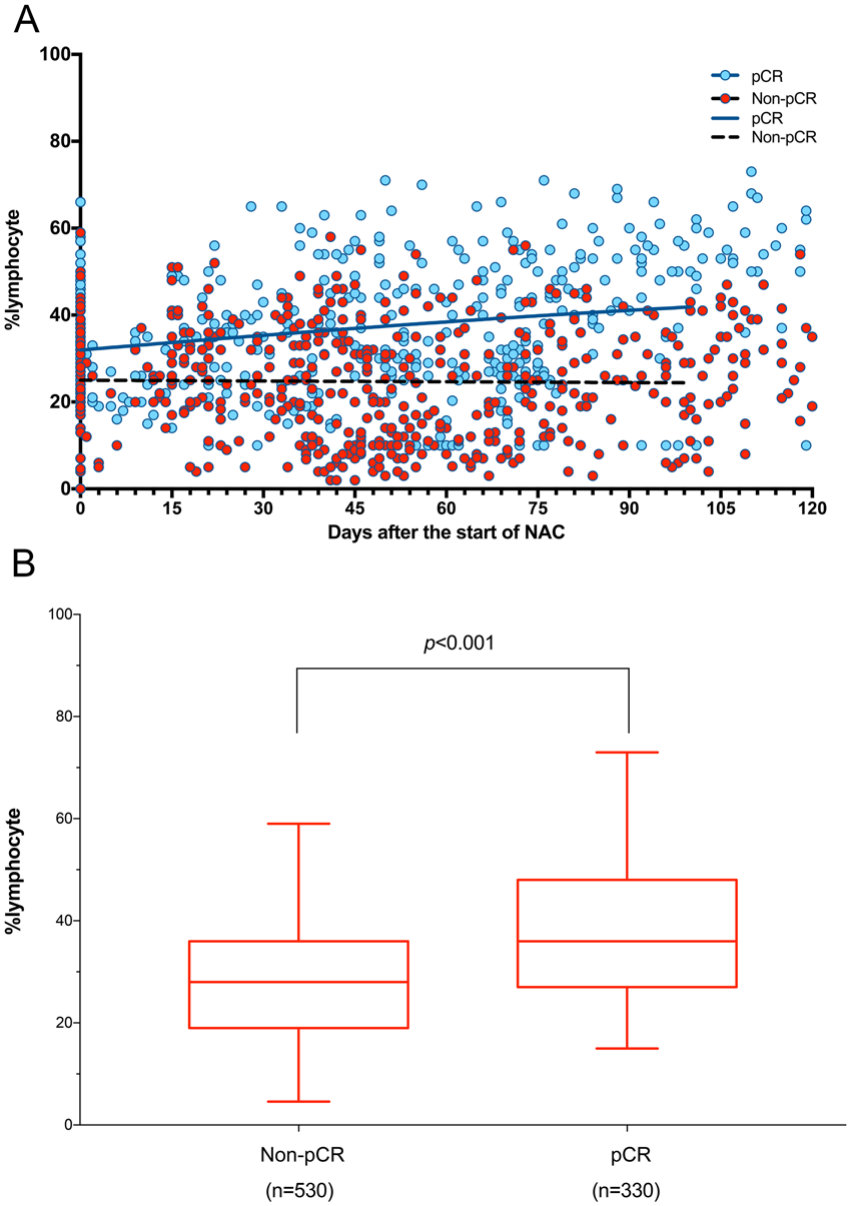
The change of circulating lymphocytes during entire NAC and the comparing between pCR and non-pCR cases.

**Figure 5:**
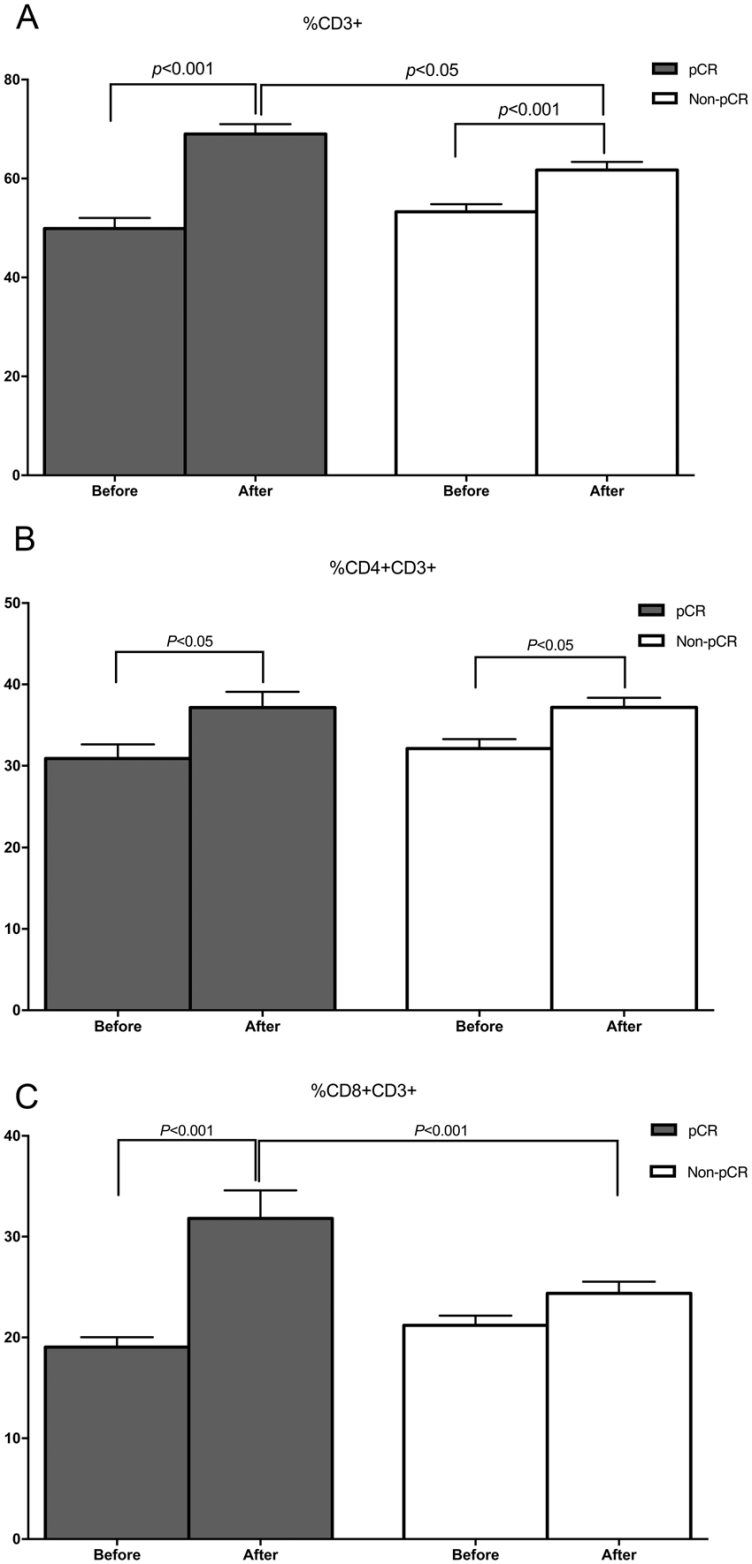
CD3+ (**A**), CD4+CD3+ (**B**) and CD8+CD3+ (**C**) ratios in circulating blood taken before and after NAC in pCR and non-pCR cases.

Subsequently, we examined the changes in the white blood cell subpopulation, including the ratios of monocyte and neutrophil and the count of platelet, during and after NAC until surgery. Since this was a retrospective study and the timing and frequency of blood tests were widely different from each patient, we plotted all the data of the entire patents according to the days from the initiation of NAC. The count of platelet tended to be slightly reduced during NAC (Fig. 3A) in the two groups, while the ratio of monocyte increased slightly during NAC and then gradually went down to the time of surgery (Fig. 3B). The proportion of neutrophil remained relatively stable during the treatment period (Fig. 3C) in both groups. In contrast, the ratio of circulation lymphocyte was markedly increased during NAC in the pCR group rather than non-pCR group (24.72 ± 0.56 vs. 35.51 ± 0.72, p < 0.001) (Fig. 4A,B). Consequently, the subsets of lymphocyte, including the percentages of CD3+, CD4+ CD3+, and CD8+ CD3+ lymphocytes, were examined before and after NAC among all patients in two groups (Fig. 5). Compared with the counterparts before NAC, the percentages of CD3+ (Fig. 5A) and CD4+CD3+ lymphocytes (Fig. 5B) were significantly increased after NAC in both groups (CD3+: pCR group 68.98 ± 13.32 vs. 49.89 ± 14.15, non-pCR group 61.73 ± 13.37 vs. 53.28 ± 12.39, *p* < 0.001 and CD4+CD3+: pCR group 37.16 ± 12.85 vs. 30.89 ± 11.56, non-pCR group 37.17 ± 9.77 vs. 32.12 ± 9.42, *p* < 0.001, respectively), while the percentage of CD8+CD3+ lymphocyte (Fig. 5C) was dramatically elevated only in pCR group (31.81 ± 18.38 vs. 19.04 ± 6.53, *p* < 0.001). Meanwhile, a higher growing of CD3+ and CD8+CD3+ lymphocyte percentages was spotted in pCR group than in non-pCR group after NAC (CD3+: 68.96 ± 13.32 vs. 61.74 ± 13.37, *p* < 0.05; CD8+CD3+: 31.81 ± 18.38 vs. 24.38 ± 9.52, *p* < 0.001, respectively).

## Discussion

NAC is considered the standard treatment for patients with AGC, providing a theoretical advantage over adjuvant chemotherapy^22,23^. Unfortunately, the overall response rate for AGC patients to NAC is still disappointed, and the pCR event after NAC is very rare around the world^10^. More recently, we clarified that the pCR rate for patients with AGC underwent NAC was approximate 17.7%, which was higher than other reports (8.4–17.4%)^13,14,24–26^. However, factors predicting the efficacy of NAC, which would be essential for the optimal management of patients with AGC, have yet to be fully elucidated. Thus, we hypothesized that blood cell counts and their subsets, which presumably reflect host immune condition, may critically affect responsiveness to NAC and OS in these patients.

In the present study, we first demonstrated the positive correlation between the pCR rate and the high levels of circulating lymphocyte ratio before and after NAC in patients with AGC. It is believed that lymphocyte, which possesses potent anticancer activities that are able to inhibit growth and metastasis in several tumors including gastric cancer, plays critical roles in host immune responses and anti-tumor immunity^27^. Kitayama *et al*. indicated that peripheral blood lymphocyte has a significant impact on the complete response to radiotherapy or neoadjuvant chemoradiotherapy (NCR) in rectal cancer patients and the maintenance of circulating lymphocyte number may improve the response to NCR in rectal cancer^28,29^. It is thus reasonable to speculate that the lymphocyte-mediated immune response against damaged tumor cells is crucial for achieving pCR after NAC in patients with AGC.

We also showed that NAC could influence the immune responses in the host, including the increasing of the ratio of lymphocyte and its subpopulation. Several *in vivo* studies have suggested that cancer cells, dead or dying due to radiotherapy and/or chemotherapy, can present tumor-associated antigens to host immune cells and thereby evoke anti-tumor immune responses^30,31^. This could be a reasonable explanation for the results in the present study that the lymphocyte ratio was dramatically increased during NAC in pCR group rather than that in the non-pCR group, although NAC is historically regarded as detrimental to immunity because of its myelosuppressive effects. Furthermore, this change has been demonstrated in other vitro studies. A significant proliferation and differentiation of the immune cells was observed after the activation of them (initial stage), and the differentiated lymphocytes are occasionally joined as two or three cells unit to perform the unique function, searching for and then attaching to tumor cells to prevent their outgrowth. These immune cells are also actively involved in the immune surveillance to avoid the metastasis of the tumor^32^.

Most recently, several studies have indicated that low lymphocyte-to-white blood cell ratios^33^, low lymphocyte-to-monocyte ratios^34^, high platelet-to-lymphocyte ratios (PLR)^21^, and increased neutrophil-to-lymphocyte ratios (NLR)^35,36^ were associated with the poor prognosis in patients with AGC. And the similar results were also observed in the present study. Before NAC, patients with low NLR (<2.2) had significantly better outcome in OS (48 vs. 28 months, *P* < 0.001) (Supplementary Figure 1), and longer OS time was found as well among patients with low PLR (<696) (36 vs 25 months, *P* < 0.001) (Supplementary Figure 2). Besides, we also revealed that the ratio of lymphocyte increased gradually during NAC, and other subsets of blood cells stabilized at the same levels compared with their counterparts before NAC. Thus, the combination of the conclusions from these studies and our results may convince our hypothesis indirectly that lymphocyte should be the key point in all these changes, and the increasing of its ratio would be positively associated with pCR and improve OS in advanced gastric cancer.

Consequently, we examined the subsets of lymphocyte to illuminate which part was the primary factor for the increased lymphocyte ratio. Our results indicated that the percentages of CD8+CD3+ and CD3+ lymphocytes were dramatically increased during NAC, but the correlation between them and the pCR event was not confirmed by multivariate analysis, except the ratios of lymphocyte before and after NAC. Theoretically, chemotherapy-induced transient lymphopenia can stimulate the production of more tumor-specific T cells, thereby eradicating inhibiting regulatory T cells, which result in an increased CD8+CD3+ cells tumor homeostasis and activity^37,38^. Thus, the influence on the pCR rate and OS may be caused by the co-work of CD3+ and CD8+CD3+ lymphocytes, which reflects the increase in the circulating lymphocyte ratio.

Also, we revealed that OS was significantly improved in patients with high lymphocyte ratios both before and after NAC. Recently, Feng *et al*. determined that the low lymphocyte ratio and the high monocyte ratio were each predictive of a poor prognosis in stage II/III gastric cancer patients receiving radical D2 gastrectomy, and exhibited greater prognostic value when considered in combination^33^. A meta-analysis by Gu *et al*. showed that the elevated platelet to lymphocyte ratio could be a significant prognostic biomarker for poor OS in patients with gastric cancer^21^. Therefore, the improvement of OS in patients with AGC receiving NAC could be partly due to an ameliorated immune response.

We acknowledge that the retrospective evaluation of the immune cell population is a limitation of our study, and the potential calculation bias cannot be excluded. However, the significant association between the circulating lymphocyte ratio and the pCR rate supports the hypothesis that total eradication of tumor cells after NAC for AGC is dependent, at least in part, on host immune reaction. Enhancing lymphocyte-mediated immunity during chemotherapy may be a lead to the improvement of the clinical efficacy of NAC in AGC patients. Our observations warrant validation in large, independent cohorts.

In conclusion, the high ratios of circulating lymphocyte, both before and after NAC, have significant impacts on chemosensitivity and improve the OS in patients with AGC, which may be considered as new prognostic biomarkers.

## Materials and Methods

### Patients

This study was performed at a single institution using a retrospective design. From January 2005 to December 2011, patients with AGC who received NAC, at the department of general surgery, Jinling Hospital, were reviewed from our database. The eligibility criteria were: 1) histologically proven gastric cancer, 2) full-text clinical record, 3) surgical resection following NAC for AGC, 4) no prior anti-tumor therapy. All cases were diagnosed by endoscopic biopsy and evaluated by contrast-enhanced computed tomography (CT) scan and endoscopic ultrasound. Laparoscopy combined with peritoneal cytology was performed in patients with potentially liver and peritoneal metastases. The TNM classification of malignant tumors 7^th^ (TNM 7^th^)^39^ is used for preoperative staging, and the number of lymph node stations was determined according to JCGC (Japanese classification of gastric carcinoma, 3rd English edition)^40^. pCR was defined as no tumor cells were detected at both the primary site and regional lymph nodes on pathological examination.

This study was approved by the Ethics Committee of Jinling Hospital according to the provisions of the Declaration of Helsinki in 1995 (as revised in Edinburgh 2000)^41^, and written informed consent was obtained from the patients before both NAC and surgery. All patients were followed up by phone call or SMS and explained clearly that data collected will be intended for publication. There were no experimental animals or human participants included in the current study. All methods were performed in accordance with the relevant guidelines and regulations.

### Treatment protocol and follow-up

All patients received 2 cycles of NAC with a regimen of 5-Fu/leucovorin/etoposide/oxaliplatin/epirubicin (FLEEOX) combination via intravenous and intra-arterial administration. 5-Fu (370 mg/m^2^) and leucovorin (200 mg/m^2^) were administered by intravenous infusion on day 1–5. Intra-arterial administration of etoposide (80 mg/m^2^), oxaliplatin (80 mg/m^2^) and epirubicin (30 mg/m^2^) was performed by Seldinger method on day 6 and 20, and the catheter was inserted through femoral artery into the celiac artery and the chemicals were injected initially at relatively high doses, followed by 14 days’ rest. The chemotherapeutic response was evaluated using contrast-enhanced CT scan by two experienced radiologists, who were blinded to any of the clinical data independently, according to the Response Evaluation Criteria in Solid Tumors (RECIST) guidelines 1.1^42^.

The patients evaluated as resectable underwent gastrectomy with D2 lymphadenectomy. D2+ lymphadenectomy would be performed if preoperative enhanced CT scan shows N3 lymph node metastases.

After surgery, all patients received 6 cycles adjuvant chemotherapy with the regimen of XELOX, oxaliplatin (130 mg/m^2^) on day 1 and xeloda (1000 mg/m^2^) on days 1 to 14 of a 28-day cycle. Patients who were subjected to recurrence would change to other regimens including adjuvant radiotherapy and/or best supportive care. For adjuvant radiotherapy, blood routine test, hepatic and renal function test and CT scan were performed before radiotherapy. Total doses ranged from 45 to 51 Gy (median 48 Gy).

All patients were followed every 3 months after adjuvant chemotherapy according to the institutional protocol. Tumor markers including CEA, CA19-9 were examined every 3 months. Chest X-ray and abdominal/pelvic enhanced CT scan were performed every 6 months. Gastroscopy was also required each year. Positron emission tomography computed tomography scan was suggested when recurrence was suspected.

### Blood cell counts and lymphocyte subsets

Venous blood samples (20 ml) were drawn into heparinized tubes from patients pre- and post-chemotherapy. The absolute white blood cell and the ratios of neutrophils and lymphocyte were determined with an automated cell counter (Beckman LH750, USA). To assess the cellular immunity, peripheral blood mononuclear cells were measured by flow cytometry (BD Biosciences, San Jose, CA) and were analyzed using CellQuest software (BD Biosciences). Cells were stained with fluorescein-labeled monoclonal antibodies: CD3-allophycocyanin, CD4-fluorescein isothiocyanate, and CD8-phycoerythrin (BD Pharmingen, San Diego, CA).

### Statistical analysis

Statistical analyses were performed using SPSS version 24.0 for MAC (SPSS Inc., Chicago, IL, USA) and GraphPad Prism version 7.0 for MAC (GraphPad Software Inc., San Diego, CA, USA). The associations of pCR with blood cell counts and various other clinical parameters were examined using Wilcoxon’s test and the chi-squared test, respectively. Multivariate stepwise logistic regression analysis was performed to determine the independence of all variables identified as possibly significant. Before and after NAC changes of the factors within each group were tested using a paired t-test. OS was calculated from the date of cases registered to the date of death or date was last known alive. Survival curves were calculated using the Kaplan-Meier method and compared by the log-rank (Mantel–Cox) test. The association of clinical factors with pathological response to NAC was assessed using logistic models. *P* values < 0.05 were considered to be significant.

### Data available statement

The datasets generated during and/or analysed during the current study are available from the corresponding author on reasonable request.

### Ethics approval and consent to participate

The ethics committee of the Medical School of Nanjing University (2009028). Chinese Clinical Trials Registry Number: ChiCTR-TRC-12002046. All patients were followed up by phone call or SMS and explained clearly that data collected will be intended for publication. All methods were performed in accordance with the relevant guidelines and regulations.

## Electronic supplementary material

Supplementary Information
