# Supplementary material for: Clinicopathological and prognostic significance of high circulating lymphocyte ratio in patients receiving neoadjuvant chemotherapy for advanced gastric cancer
Source: Sci Rep. 2018 Apr 18;8:6223. doi: 10.1038/s41598-018-24259-5 (PMC5906667; doi:10.1038/s41598-018-24259-5)
Supplement: Supplementary file 1 — Supplementary Information [file 41598_2018_24259_MOESM1_ESM.pdf]

Title page

Title: Clinicopathological and prognostic significance of high circulating lymphocyte ratio in patients receiving neoadjuvant chemotherapy for advanced gastric cancer

Authors

Yang Li[1,2], Yao Wei[3], Qi He[2], Xulin Wang[2], Chaogang Fan[2] and Guoli Li[2]

Author Affiliations

1. Division of Digestive Surgery, Xijing Hospital, Fourth Military Medical University, 127 West Changle Road, 710032, Xi'an, Shaanxi, China

2. Research Institute of General Surgery, Jinling Hospital, School of Medicine, Nanjing University, 305 Zhongshan Eastern Road, Nanjing 210002, China

3. The First Affiliated Hospital of Soochow University, Department of Medicine, Emergency and Critical Care Medicine, Suzhou 215003, China

Yang Li: li_yang82@icloud.com

Yao Wei: dr_betty@126.com

Qi He: xiaoran8210@gmail.com

Xulin Wang 54645121@qq.com

Chaogang Fan: fanchaogangmed@126.com

Guoli Li: liguoli82@126.com

Correspondence author

Guoli Li, Ph.D., Research Institute of General Surgery, Jinling hospital, 305 Zhongshan East Road, Nanjing, 210002, China. Tel: 86-25-80863334; Fax: 86-25-84803956. Email: liguoli82@126.com

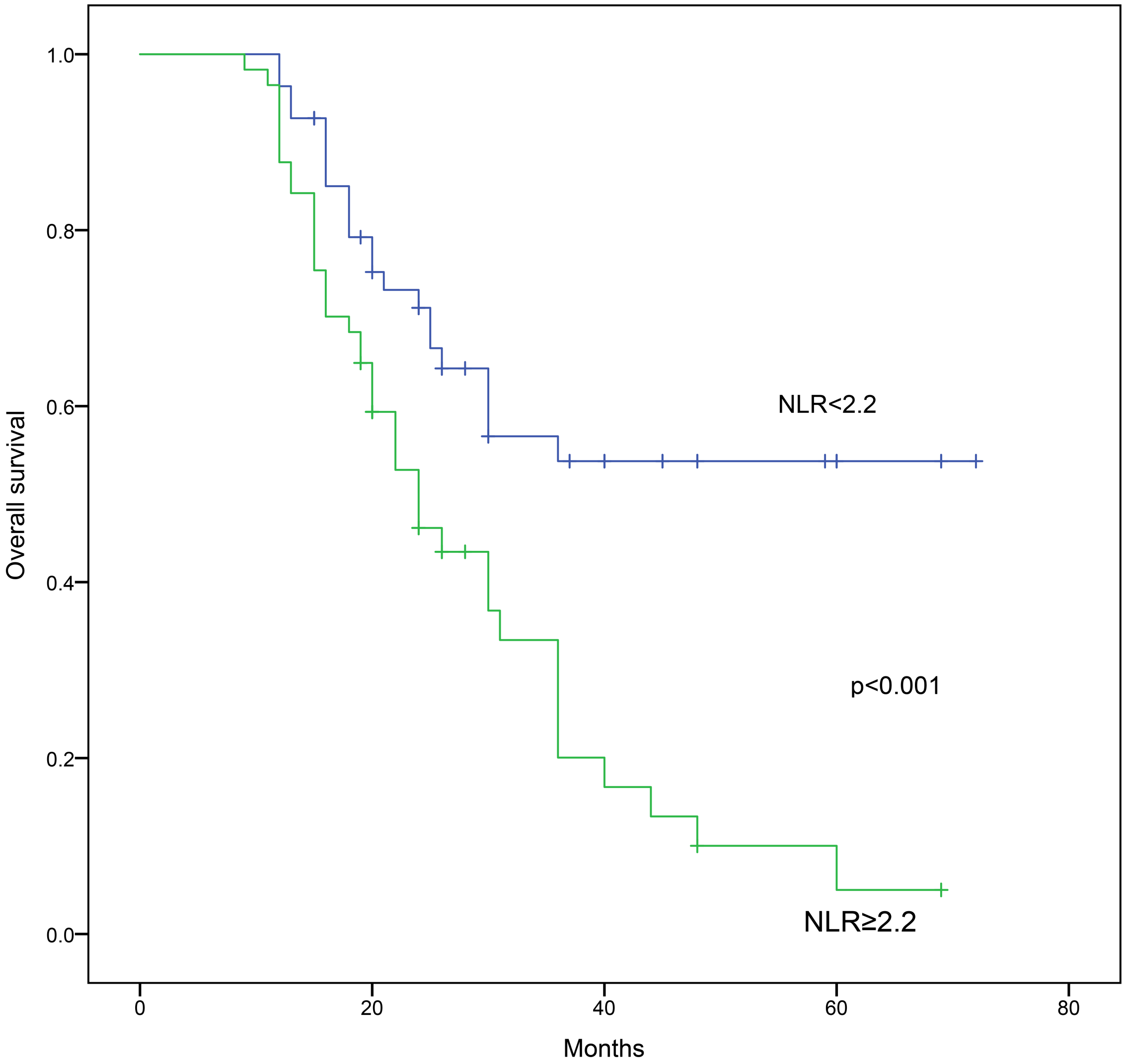

Supplementary Figure 1. Overall survival of the patients with high and low NLR taken before NAC.

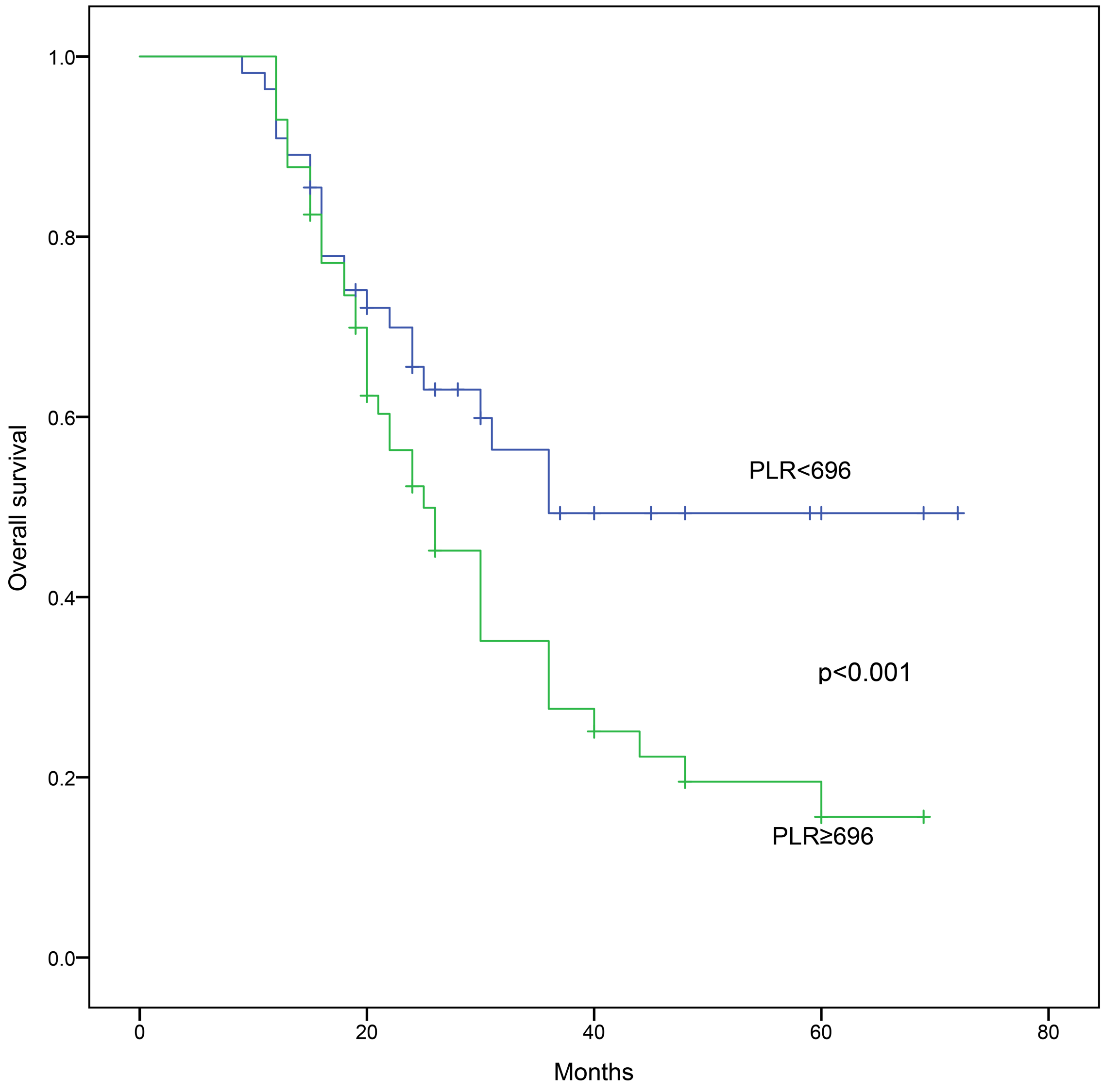

Supplementary Figure 2. Overall survival of the patients with high and low PLR taken before NAC.
